# Research on evaluation indicators of healthspan for veterans: A single-center exploratory study

**DOI:** 10.12669/pjms.40.7.8714

**Published:** 2024-08

**Authors:** Jianbo Zhu, Jingda Zhang, Tongbo Liu, Yong Zhang, Guogang Xu

**Affiliations:** 1 Jianbo Zhu Health Management Institute, The Second Medical Center & National Clinical Research Center for Geriatric Diseases, Chinese PLA General Hospital, Beijing, 100853, China; 2 Jingda Zhang Health Management Institute, The Second Medical Center & National Clinical Research Center for Geriatric Diseases, Chinese PLA General Hospital, Beijing, 100853, China; 3 Tongbo Liu Department of Information, Medical Supplies Center, Chinese PLA General Hospital, Beijing, 100853, China; 4 Yong Zhang Dept. of Oncology, The Second Medical Center and National, Clinical Research Center for Geriatric Diseases, Chinese PLA General Hospital, Beijing, 100853, China.; 5 Guogang Xu Health Management Institute, The Second Medical Center & National Clinical Research Center for Geriatric Diseases, Chinese PLA General Hospital, Beijing, 100853, China

**Keywords:** Veterans, Healthspan, Health assessment

## Abstract

**Objective:**

To explore a definition of healthspan that based on actual situation of veterans is of significance for improving their health status and life quality.

**Methods:**

This was a retrospective study. Based on the medical data of veterans from the Chinese PLA General Hospital. Total of 1,421 subjects were enrolled to this study, among which 441 deceased cases were further analyzed. The indicators of healthspan of the subjects was calculated from four dimensions (the status of chronic diseases, physical function, social function and psychological function). The risk factors for death were analyzed in a population cohort from 2008 to 2021 (including 763 subjects, among which 372 were deceased).

**Results:**

The average lifespan and adjusted healthspan of the subjects were 93.3 years and 75.1 years, respectively. The four dimensions of healthspan were: adjusted healthspan without chronic diseases was 76.3 years, social function-related healthspan was 88.8 years, physical function-related healthspan was 91.5 years, and psychological function-related healthspan was 92.7 years. By analyzing the cohort in 2008, it was inferred that the main risk factors for the death of veterans were poor nutritional status, renal function injury, high blood pressure, high blood sugar, and aging.

**Conclusions:**

This study proposed four dimensions related to “healthspan” for Chinese veterans (adjusted healthspan without chronic diseases, physical function-related healthspan, social function-related healthspan, and psychological function-related healthspan). Besides, poor nutritional status, renal function injury, and high blood pressure were the most important risk factors affecting the death of veterans.

## INTRODUCTION

With the increasing aging of the population, it has become a consensus that longevity is not simply equivalent to health. The World Health Organization (WHO) has begun to use the healthspan^1^ to reflect the health status of the population since 1997, which is a comprehensive indicator that reflects the quality of life and is combined with the lifespan of the population to reflect the health level of the population. However, the WHO has not yet proposed a clear definition or scientific standards for individual “healthspan”. Currently, the definition and measurement methods of healthspan varies from different definitions of health status, but most of them are based on multiple aspects such as physiological status, psychological status, and social functional status.^2^

For veterans whose lifestyles and medical interventions differ from ordinary elderly people significantly, they are more prone to problems such as military training injuries, post-traumatic stress disorder, depression, and anxiety.^3^,^4^ Proposing a definition of healthspan that based on the actual situation of veterans is of significance for improving their health status and their life quality.^5^,^6^

## METHODS

This was a retrospective study. A total of 1,421 subjects who received medical treatment at the Chinese PLA General Hospital were included, among which 441 were deceased, and the characteristics of their disease were analyzed. The healthspan of the subjects was calculated from four dimensions: adjusted healthspan without chronic diseases, physical function-related healthspan, social function-related healthspan, and psychological function-related healthspan. The risk factors for death were analyzed using a population cohort from 2008 and followed up to 2021 (including 763 subjects, among which 372 were deceased).

### Ethical Approval:

The study was approved by the Institutional Ethics Committee of Chinese PLA General (No..: S2021-095-02; date: April 09, 2021).

Definition of healthspan: Based on previous research results^7^,^8^ and the health characteristics of Chinese veterans, the definition of healthspan should include four dimensions: adjusted healthspan without chronic diseases, physical function-related healthspan, social function-related healthspan, and psychological function-related healthspan. The standards are set as follows:

***(1) Adjusted healthspan without chronic diseases:*** The occurrence of chronic diseases is used as the endpoint for the adjusted healthspan without chronic diseases. The chronic diseases involved in this study include hypertension, heart disease/coronary heart disease, diabetes, cerebrovascular disease, chronic bronchitis/COPD, and malignant tumors. Considering that veterans receive better medical interventions after the diagnosis of chronic diseases, and their indicators and functions can be maintained in a good state under medication and other means of intervention, the adjusted healthspan without chronic diseases is further defined as: acute recurrence of the above-mentioned chronic diseases, or the appearance of related complications, or the recurrence and metastasis of malignant tumors are determined as the end.

***(2) Physical function-related healthspan:*** Physical function is the basic ability of daily life. Physical function-related healthspan is determined by the appearance of diseases or medical interventions that affect daily life and activity ability. It includes joint system diseases that affect daily activities, Parkinson’s disease, the need for wheelchair assistance or bed rest, and the use of catheters such as indwelling urinary catheters and gastrointestinal tubes.

***(3) Social function-related healthspan:*** Social communication ability includes visual, auditory, and language abilities, as well as a certain level of intelligence. Social function-related healthspan is determined on the appearance of visual/ auditory/language disorders, or dementia.

***(4) Psychological function-related healthspan:*** The endpoint is the occurrence of unhealthy psychological status such as anxiety, depression, and bipolar emotional disorders. The above four dimensions are the basic elements for healthspan, and the minimum criterion among the four dimensions is the adjusted healthspan.

### Statistical analyses:

R 4.3.0, Rstudio, and JASP 16.0.0 software were used for data sorting and analysis. The mean value and 95% confidence interval (CI) were used to describe each dimension of healthspan. The distribution characteristics of different indicators were described according to survival and death, and the differences were tested by students’-test. The multifactor COX equal proportional risk model was used to explore the influencing factors for the death of the population cohort from 2008 to 2021.

## RESULTS

As of August 2021, there were 441 deceased subjects in total, with an average lifespan of 92.31±7.70 years. The ages of death mainly ranged from 90 to 99 years, accounting for 67.35% of all subjects. The primary causes of death were malignant tumors (156, 35.37%), respiratory diseases (151, 34.24%), cardiovascular diseases (63, 14.29%), followed by cerebrovascular diseases, urinary diseases, digestive diseases, endocrine diseases and trauma.

Among them, the top three accounted for more than 80%. The main cause of death for those under 90 years old was malignant tumors. While the proportion of deaths caused by respiratory diseases gradually increased with age growing, especially for those over 90 years old, respiratory diseases gradually became the main cause of death. The prevalence of chronic diseases, including hypertension, heart disease, diabetes, cerebrovascular disease, COPD, urinary system diseases, and tumors were all higher than 50%. Among them, the prevalence rate of heart disease was the highest of 90.93%, while the prevalence rate of ophthalmic diseases was the lowest of 7.03%. The comorbidity rate was 99% (439/441 cases), and the proportion of those with five or more diseases was 82.31% (363/441 cases).

The healthspan without chronic diseases for the deceased subjects was 61.6 years (95% CI: 60.2-63.0), and the adjusted healthspan without chronic diseases was 76.3 years (95% CI: 75.1-77.4). The physical function-related healthspan was 91.5 years (95% CI: 90.7-92.3), the social function-related healthspan was 88.8 years (95% CI: 87.8-89.7), and the psychological function-related healthspan was 92.7 years (95% CI: 92.1-93.3). The adjusted healthspan was 75.1 years (95% CI: 73.9-76.3) according to data in this study (Table-I).

**Table-I:** Average Levels of lifespan and healthspan.

		Standard deviation	95% Confidence Interval	

			Lower limit	Upper limit
Lifespan	93.38	0.27	92.84	93.92
Adjusted healthspan without chronic diseases	76.27	0.59	75.11	77.43
Physical function-related healthspan	91.55	0.38	90.80	92.30
Social function-related healthspan	88.80	0.47	87.89	89.72
Psychological function-related healthspan	92.77	0.30	92.20	93.35
Adjusted Healthspan	75.16	0.62	73.94	76.39

Based on clinical information of 441 deceased subjects and 980 living subjects as the data source, four population cohorts in 2008, 2013, 2018, and 2021 were formulated. The most representative 2008 population cohort was selected for further analysis. A total of 763 study subjects were included in the study. As of September 2021, a total of 372 subjects died, and the mortality rate was 48.8%. For the deceased subjects by 2021, the levels of white blood cell, urea, serum creatinine, plasma D-dimer, and brain natriuretic peptide precursor were higher than those of the surviving subjects (P < 0.05), while the levels of BMI, systolic blood pressure, diastolic blood pressure, total cholesterol, low-density lipoprotein cholesterol, high-density lipoprotein cholesterol, hemoglobin, pre-albumin, and serum albumin were lower than those of the survivors (P < 0.05). (Table-II)

**Table-II:** Functional indicators of the 2008 cohort for follow-up outcome (death) in 2021.

	T	df	Survivors	Deaths	p
Gamma glutamyl transferase	-1.063	658	32.41±37.17	35.65±39.87	0.288
White blood cell count	-3.465	657	5.97±1.79	6.55±2.32	<0.001
Alanine aminotransferase	3.531	654	22.26±28.76	16.38±12.32	<0.001
Amylase	-1.65	615	70.03±25.81	74.64±39.01	0.1
Carbon dioxide	-0.766	655	25.22±2.17	25.37±2.55	0.444
Red blood cell count	13.36	657	4.48±0.5	3.93±0.55	<0.001
Troponin I	0.219	356	0.44±4.09	0.34±4	0.827
Myoglobin quantification	-0.883	377	59.11±77.86	111.98±616.88	0.378
Potassium	-1.908	643	4.01±0.31	4.07±0.39	0.057
Alkaline phosphatase	-1.034	657	62.19±23.42	64.39±29.7	0.301
Chloride	2.391	655	103.99±3.03	103.34±3.77	0.017
Sodium	5.049	655	141.72±2.86	140.32±3.96	<0.001
Urea	-7.407	656	5.9±1.82	7.72±3.85	<0.001
Glucose	-0.812	656	5.71±1.5	5.81±1.82	0.417
Lactate dehydrogenase	-1.354	652	162.8±36.45	170.53±90.82	0.176
Aspartate aminotransferase	0.439	658	22.44±27.41	21.61±21.13	0.661
Plasma D-dimer determination	-6.078	489	0.43±0.48	1.06±1.29	<0.001
Plasma Fibrinogen Determination	-6.691	613	3.1±0.67	3.62±1.11	<0.001
Serum uric acid	0.801	658	336.24±76.51	330.52±100.86	0.424
Platelet count	-0.063	657	180.52±49.74	180.8±61.72	0.95
Lipase	-0.694	396	110.81±84.24	119.85±141.78	0.488
Direct bilirubin	-0.017	656	3.59±1.89	3.6±4.99	0.987
Total bilirubin	3.179	657	12.62±5.39	10.76±8.71	0.002
Total protein	4.252	656	70.99±5.7	68.99±6.19	<0.001
Creatinine	-5.71	610	79.89±18.73	94.88±40.89	<0.001
Hemoglobin determination	15.151	608	144.46±15.93	124.9±15.91	<0.001
Serum albumin	13.804	610	42.38±3.59	38.51±3.34	<0.001
LDL cholesterol	7.341	599	2.62±0.65	2.22±0.68	<0.001
Triglycerides	0.936	595	1.51±0.74	1.45±0.82	0.35
HDL cholesterol	3.613	599	1.26±0.32	1.16±0.34	<0.001
Total cholesterol	6.524	599	4.43±0.86	3.98±0.84	<0.001
Pro- brain natriuretic peptide	-2.403	340	420.52±1504.87	832.63±1368.9	0.017
Prealbumin assay	6.624	340	26.58±6.42	21.82±5.94	<0.001
Determination of global glycated hemoglobin	-4.749	305	6.03±0.74	6.46±0.87	<0.001
Reticulocyte count	-1.479	154	1.39±0.42	1.55±0.66	0.141
Breathe	-3.19	492	18.06±0.59	18.38±1.22	0.002
Pulse	-0.872	492	71.25±7.34	71.91±8.12	0.384
Body temperature	-1.802	492	36.26±0.44	36.34±0.52	0.072
Height	0.889	486	168.08±18.52	166.49±19.04	0.374
Weight	7.716	492	75.26±19.72	55.36±30.35	<0.001
High blood pressure	-3.051	492	126.05±12.04	130.21±15.45	0.002
Low blood pressure	3.154	492	71.15±9.63	68.21±9.91	0.002

Multivariate analysis and generalized additive model analysis of the 2008 cohort showed that hypertension, renal dysfunction, and high plasma D-dimer were the most important risk factors, while high hemoglobin levels may reduce the risk of mortality in this population (Table-III).

**Table-III:** Multivariate analysis of the 2008 cohort for follow-up outcome (death) in 2021.

	Estimate	Standard deviation	Risk ratio	P	95% CI lower limit	95% CI upper limit
Hypertension	2.822	0.748	16.812	<0.001	3.877	72.901
Dyslipidemia	0.058	0.307	1.06	0.85	0.58	1.936
Diabetes	0.194	0.31	1.214	0.531	0.661	2.23
Renal dysfunction	1.33	0.541	3.781	0.014	1.31	10.909
Liver function abnormality	-0.385	0.356	0.68	0.279	0.339	1.366
Plasma D-dimer measurement	0.743	0.277	2.102	0.007	1.222	3.616
Hemoglobin measurement	-0.048	0.011	0.953	<0.001	0.933	0.974
Brain natriuretic peptide precursor	0	0	1	0.912	1	1
White blood cell count	-0.003	0.068	0.997	0.961	0.871	1.14
BMI	-0.008	0.005	0.992	0.111	0.981	1.002

The risk factors with statistical significance (P<0.01) in the above analysis can be classified according to physiological functions: high blood pressure (SBP, DBP), high blood lipids (TC, LDL-C, HDL- C), high blood sugar (Hb1A1c), abnormal liver function (ALT, TBil), abnormal renal function (BUN, Cr), poor coagulation function (Fib, D-dimer), and poor nutritional status (Hb, TP, Alb, P -Alb). The above research showed that the death risks mainly include: poor nutritional status, renal function injury, high blood pressure, high blood sugar, and age (Table-IV). Among them, the first three were the most important factors, and more attention should be paid on these aspects to improve life quality.

**Table-IV:** Functional classification of the 2008 cohort for follow-up outcome (death) in 2021.

	Estimate	Standard error	Hazard ratio	P value	95% CI Lower limit	95% CI upper limit
Age	0.109	0.032	1.115	0.001	1.047	1.187
Hypertension	1.300	0.232	3.670	0.000	2.330	5.780
Dyslipidemia	0.016	0.182	1.017	0.928	0.712	1.452
Diabetes	0.603	0.179	1.827	0.001	1.287	2.593
Renal insufficiency	1.560	0.330	4.761	0.000	2.494	9.088
Abnormal liver function	0.030	0.252	1.030	0.906	0.629	1.688
Coagulation	0.083	0.180	1.087	0.644	0.764	1.547
Poor nutritional status	2.226	0.269	9.263	0.000	5.464	15.704
Constant	-1.028	0.180	0.358	0.000		

## DISCUSSION

Based on the study results, the four dimensions of veterans’ healthspan in ascending order were: adjusted healthspan without chronic diseases (76.3 years), social function-related healthspan (88.8 years), physical function-related healthspan (91.5 years), and psychological function-related healthspan (92.7 years). It can be seen that the main factors affecting the healthspan were chronic diseases, which suggested that for veterans, moderate exercise, nutritional supplement, active screening and effective intervention are needed to reduce the damage of chronic diseases on the overall health.^9^,^10^

The main causes of death among veterans in our center were malignant tumors, respiratory diseases, and cardiovascular diseases, which indicated that screening and intervention for these diseases should be especially emphasized in daily medical and health care work. The average lifespan and adjusted healthspan of veterans in our center were 93.3 years and 75.1 years, respectively, which may be attributed to current proactive health care and medical strategies, apart from individual differences and differences between urban and rural medical levels. The difference between the average lifespan and adjusted healthspan was 18.2 years, which was also related to proactive medical interventions that enable veterans to survive for a long time even in a non-healthy state, greatly extending their overall lifespan.^11^,^12^

The healthspan without chronic diseases of veterans was 61.6 years, and the adjusted healthspan without chronic diseases was 76.3 years. The difference showed that after the diagnosis of the relevant chronic diseases, with the active intervention of drugs and lifestyle, the chronic diseases could be maintained under control for a long time until the occurrence of serious complications or repeated acute attacks.^13^,^14^ From the perspective of three-level prevention, the first is to prevent or delay the occurrence of chronic diseases, the second is to achieve early diagnosis and treatment of chronic diseases, and the third is to prevent the deterioration of existing chronic diseases to prevent complications and disabilities.^15^,^16^ Therefore, to further extend the healthspan of veterans, more efforts should be focused on the prevention and treatment of chronic diseases, which is also in accordance with the three-level prevention strategy.

The death risks of veterans mainly included: poor nutritional status, renal function injury, high blood pressure, high blood sugar, and aging. Studies have proved that high quality of nutrition can effectively improve body function, extend lifespan and healthspan.^17^ Patients with renal function injury are prone to depression, anxiety, physical weakness, and lack of social support,^18^-^19^ and elevated blood pressure and blood sugar lead to increased risk of multiple chronic diseases.^20^ For the health management of veterans, the monitoring and intervention of key indicators should be strengthened to improve the level of healthy aging.^21^

### Limitations of this study

There were data bias in psychological assessments due to the limitation of assessment methods. Besides, the study lacks the detailed research by age group, and it is necessary to further explore the different health status and health promotion strategies of veterans in different age groups.

## CONCLUSIONS

This manuscript conforms the Enhancing the Quality and Transparency Of health Research (EQUATOR) network guidelines. The study proposed four dimensions of healthspan of Chinese veterans (adjusted healthspan without chronic diseases, physical function-related healthspan, social function-related healthspan, and psychological function-related healthspan). Each dimension of healthspan can better reflect the overall health of the study subjects. More intervention can be carried out from the above four dimensions. By analyzing the health data of veterans, the disease spectrum was established. The study found that cancer screening, and strengthen the health management of chronic diseases such as respiratory system and cardiovascular system should be focused on for veterans under 90 years old, Furthermore, health care for those over 90 years old should especially focus on strengthening the diagnosis and treatment of respiratory system and cardiovascular system diseases and health management.

The core functional indicators affecting the death of veterans were systematically analyzed, especially the abnormal indexes of white blood cells, urea, serum creatinine, plasma D dimer, and hypertension, renal insufficiency, poor nutritional status and other important diseases. The study provided some basis for the relevant key indexes and diseases that should be paid attention to in the health care work of veterans.

### Authors’ Contributions:

**JZ** and **JZ:** Carried out the studies, data collection, drafted the manuscript, and are responsible and accountable for the accuracy and r integrity of the work.

**TL YZ GX::** Performed the statistical analysis and participated in its design.

All authors have read and approved the final manuscript.
